# Effect of a Novel Nonviral Gene Delivery of BMP-2 on Bone Healing

**DOI:** 10.1100/2012/560142

**Published:** 2012-11-11

**Authors:** P. Schwabe, S. Greiner, R. Ganzert, J. Eberhart, K. Dähn, A. Stemberger, C. Plank, G. Schmidmaier, B. Wildemann

**Affiliations:** ^1^Center for Musculoskeletal Surgery and Julius Wolff Institute, Charité-University Medicine Berlin, Campus Virchow, Augustenburger Platz 1, 13353 Berlin, Germany; ^2^Institute for Experimental Oncology and Therapy, Technical University Munich, 81675 Munich, Germany; ^3^Department for Trauma and Reconstructive Surgery, University of Heidelberg, 69118 Heidelberg, Germany; ^4^Berlin-Brandenburg Center for Regenerative Therapies, Charité-University Medicine Berlin, 13353 Berlin, Germany

## Abstract

Background. Gene therapeutic drug delivery approaches have been introduced to improve the efficiency of growth factors at the site of interest. This study investigated the efficacy and safety of a new nonviral copolymer-protected gene vector (COPROG) for the stimulation of bone healing. Methods. *In vitro*, rat osteoblasts were transfected with COPROG + luciferase plasmid or COPROG + hBMP-2 plasmid. *In vivo*, rat tibial fractures were intramedullary stabilized with uncoated versus COPROG+hBMP-2-plasmid-coated titanium K-wires. The tibiae were prepared for biomechanical and histological analyses at days 28 and 42 and for transfection/safety study at days 2, 4, 7, 28, and 42. Results. *In vitro* results showed luciferase expression until day 21, and hBMP-2-protein was measured from day 2 – day 10. *In vivo*, the local application of hBMP-2-plasmid showed a significantly higher maximum load after 42 days compared to that in the control. The histomorphometric analysis revealed a significantly less mineralized periosteal callus area in the BMP-2 group compared to the control at day 28. The rt-PCR showed no systemic biodistribution of luciferase RNA. Conclusion. A positive effect on fracture healing by nonviral BMP-2 plasmid application from COPROG-coated implants could be shown in this study; however, the effect of the vector may be improved with higher plasmid concentrations. Transfection showed no biodistribution to distant organs and was considered to be safe.

## 1. Background

Bone healing problems in terms of delayed or nonunions remain a relevant clinical problem. Advances in understanding fracture repair and biological healing of osseous tissue led to a variety of methods to stimulate the healing process. The stimulation of bone healing with application of several recombinant growth factors has moved into scientific and clinical focus. 

After discovery of the osteoinductive properties of demineralised bone matrix by Urist and Mclean in 1965 [1] rhBMP-2 and rhBMP-7 are now approved by the U.S. Food and Drug Administration (FDA) for restricted clinical use in open tibial fractures and anterior spinal fusion (rhBMP-2) or for the treatment of tibial nonunions and posterolateral lumbal arthrodesis (rhBMP-7). The clinical findings are promising and the therapy is cost effective [2], but compared to the impressive results of many experimental models, they are lacking behind [3–5]. The reason for that is still unclear, and it might raise the concern that a single exposure might eventually not lead to a sufficient osteoinductive signaling. It has been suggested that bone repair stimulated with regional gene therapy is influenced not just by the amount of protein expression but also by duration of protein production [6]. So the important issue when accessing growth factors for local bone repair maintains to identify the ideal drug delivery carrier which assures a sufficient concentration and effect of the protein at the application site for the duration of the healing process and therefore providing an appropriate support for the bony repair. A gene therapeutic approach might be the answer to that problem. 

The half-life of growth factors as part of natural regulation mechanisms is short [7]. Since gene therapy provides the gene for the protein rather than just the degradable protein, this technique might result in a higher and more constant level of the protein for a defined time period [8]. The continuous local production of these proteins might then lead to an initiation and acceleration of cellular processes resulting in enhanced fracture healing. Numerous *in vitro* and *in vivo* studies proved that appropriate cells are able to produce growth factors [9] and thereby improve bone metabolism and fracture healing after transfection with growth factor specific genes [10, 11]. 

To ferry the gene for the specific protein into the target cell a delivery vehicle (vector) is needed. In general, two types of vectors are in the focus of scientific research: viral and nonviral vectors. Viral transduction is generally considered the most efficient method available for delivering genetic material to target cells. Different modes of application like implanting gene activated matrices [7], intraoperative [11, 12] or percutaneous [13] injection of viral vectors have successfully been applied in fracture healing and bone defect healing situations [14]. 

But viral vectors could cause immune reactions, which might inhibit transgene expression [15–17]. Also, the gene sequence of some viral vectors might be integrated in the genome of the host cells, which could lead to an uncontrolled dissemination, or even cause malignant transformations. These possible limitations might limit the utility of these vectors in the field of bone healing.

In nonviral gene delivery several techniques for transfection can be used to bring genes into a target cell; these include exposing the target to naked DNA [18], using liposomes [19, 20], or by using methods like electroporation [21]. The nonviral methods of gene delivery are associated with minimal immunogenicity and consequently might be safer compared to viral methods [22]. In addition, nonviral vectors are often easier to produce than viral vectors. However, they are not as effective at delivering the desired gene into the host cell [22, 23].

The aim of this study was to investigate the effect of BMP-2 gene delivery by the new nonviral vector (COPROG) incorporated in a poly(D,L-lactide) implant coating on fracture healing. Since the safety issue is crucial in gene therapy models a main focus was on the transfection safety by excluding systemic side effects and assuring a controlled and localized gene delivery. 

## 2. Material and Methods

In this study a newly developed nonviral vector for local and controlled gene delivery for growth factors was used [24–26]. A novel protective copolymer (PROCOP) P6YE5C (prepared from polyethyleneglycol (PEG) 6000) was synthesized by coupling anionic peptides to a reactive copolymer backbone. The synthesis can be carried out by established methods of peptide chemistry. After the synthesis of the protective copolymer P6YE5C it was purified using a Superdex HighLoad XK 26/70 column (Amersham Biosciences, Freiburg, Germany). It has been shown that “PROCOPs” stabilize the DNA polyplexes in small size and protect them from complement activation and opsonisation. Both, the number and the amount of plasma proteins adsorbed to the copolymer-protected polyplexes were efficiently reduced through the shielding effect of the protective copolymers [26]. 

To form the copolymer-protected gene vector (COPROG) a preformed PEI (polyethylenimine) cDNA polyplex with excess positive charge was incubated with the negatively charged peptide-PEG copolymer (PROCOP) resulting in a surface layer of PEG loops around the cDNA polyplex. 

The Plasmids were expanded and purified by PlasmidFactory GmbH & Co. KG, Bielefeld, Germany. The plasmid p55pCMV-IVS-luc+ coding for the firefly luciferase as a reporter gene under the control of the CMV promoter was kindly provided by Andrew Baker, Bayer Corp., USA. The cDNA of human bone morphogenetic protein 2 (BMP-2) was kindly provided by Genetics Institute, Inc., Cambridge, MA, USA. The plasmid pB-BMP2 was derived from p55pCMV- IVS-luc+ by removing the luciferase-encoding sequence using the Qiagen (Hilden, Germany) gel extraction kit after Hind III/Fse I digestion and by inserting the BMP-2 coding sequence which was PCR amplified in order to introduce Hind III/Fse I restriction sites.

### 2.1. Implants and Coating Technology

Poly(D,L-lactide) (Boehringer, Germany), 30 kDa molecular weight, was solved in ethylacetate, and preassembled COPROGs were incorporated. Titanium Kirschner wires (K-wire, 1.0 mm diameter; Synthes Co., Switzerland) were coated two times and dried under sterile conditions. During this process each implant was charged with approximately 40 *μ*g of DNA. The properties of the PDLLA coating have been described elsewhere [27].

### 2.2. Plasmids

Plasmids used were p55pCMV-IVS-BMP-2 as therapeutic gene and p55pCMV-IVS-Luc+ as reporter gene.

### 2.3. Cell Culture

Primary rat osteoblasts were used. Characterization was performed with E11 antibody staining against rat osteoblasts [28]. Cells were maintained in Dulbecco's modified Eagle's medium (DMEM) containing 10% FBS, 100 U/mL of penicillin, and 100 mg/mL streptomycin. The cells were plated at a density of 100,000 cells/well in a 6-well plate. For transfection the differently coated K-wires were placed into the wells. 

For the proof of principle in the first cell culture two groups were analyzed: K-wires coated with PDLLA and (1) COPROG + luciferase plasmid (40 *μ*g) or (2) naked-luciferase-DNA plasmid (40 *μ*g).

The luciferase activity was measured at day 6, 14, 21 and 28 in the supernatant with Promega luciferase assay according to the manufacturer and with Luminometer (Berthold Technologies). Luciferase activity (RLU: relative light units) was set in relation to the total protein concentration (*μ*g), which was measured by Coomassie Protein Assay.

In a second approach the amount of human BMP-2 produced by the rat cells was measured in the supernatant after adding K-wires coated with COPORGs + BMP-2 plasmid (40 *μ*g) to the cells. K-wires coated with the copolymer without plasmid served as control group. Human BMP-2 was measured with ELISA (R&D Systems) according to the manufacturer at day 2, 4, 6, 8, 10, and 12. 

### 2.4. Animals and Fracture Model

Five-month-old female Sprague Dawley rats (mean body weight 250 g) (Harlan-Winkelmann, Germany) (*n* = 145) were used. Sedation was achieved with Ketamine hydrochloride (100 mg/mL) (80 mg/kg body weight) and Xylazin 2% (12 mg/kg body weight). The cortical bone and medullary canal were opened using a 1 mm steel K-wire. After removing the K-wire the tibia and fibula were fractured with a standardized fracture device [29]. Following closed reduction the tibiae were intramedullary stabilized with either coated or uncoated titanium K-wires. All experiments were approved by the Animal Experimentation Ethics Committee of Berlin. Following groups were investigated (Table 1).

### 2.5. Radiographic Evaluation

Biplane radiographs (posterior-anterior and lateral view) were taken immediately after surgery to ensure the correct position of the intramedullary wire and to exclude bone displacements after the stabilization procedure. Additionally X-rays were taken at each followup (day 7, 14, 21, and 28). All X-rays were recorded digitally (Fujifilm IP-cassette, Fuji) using a Mobilett Plus X-ray unit (Siemens AG, Germany). 

### 2.6. Systemic Parameters

At days 0, 7, 14, 21, and 28 blood and serum samples (0.5 mL) were taken from the ophthalmic vein plexus from 10 animals each group. The blood samples were analyzed on routine laboratory parameters. Additionally rectal body temperature was measured, and body weight was determined. 

### 2.7. Mechanical Testing

At days 28 and 42 the 10 animals of each group were sacrificed. Both tibiae were harvested and the surrounding soft tissue was thoroughly removed. Before biomechanical torsional testing the wires were carefully explanted without damaging the fracture callus. The proximal and distal ends of the tibiae were embedded with bone cement (Beracryl, Fa. Troller, Switzerland) and placed into a testing device. The bone was preloaded by an axial force and a constant linear propulsion (*v* = 2 mm/min) was applied by the testing machine (Model 1455, Zwick, Germany). The translation of the material testing machine was transformed to a uniform torsional movement. The torsional stiffness and maximum load of the fractured tibiae were calculated and compared to the nonfractured contralateral tibiae.

### 2.8. Bone Histomorphometry

For bone histomorphometry 10 fractured tibiae of each group were harvested 28 and 42 days after surgery. After liberating the bone from the soft tissue the implanted K-wire was carefully pulled out of the intramedullary canal. The specimens were fixed for two days in 10% normal buffered formaldehyde followed by dehydration in ascending ethanol concentrations and embedded in methylmethacrylate (Technovit 9100, Heraeus Kulzer, Germany). Using a microtome (Leica, Germany) 5 *μ*m longitudinal sections was cut and stained with v. Kossa and with Safranin O/Light Green. Histomorphometrical parameters were measured with a microscope (Leica, Germany) and the Zeiss KS 400 image analyzing system (Zeiss, Germany) as described previously [30]. Briefly, the callus was divided into the proximal and distal part, and a 1.5 length of the tibial diameter was used to define the ROI of proximal and distal callus halves. The total diameter of the callus was included in the ROI. To draw a histomorphological comparison between the groups, the mineralized area of the cortices, the area of the periosteal callus, and the mineralized and cartilaginous volume of the callus were measured.

### 2.9. PCR Analysis

At days 2, 4, 7, 28, and 42 days after surgery 5 animals of the luciferase-group IV were sacrificed and tested for luciferase transfection. 

Besides the complete right tibia seven other tissues (brain, lung, liver, spleen, kidney, ovaries, ipsilateral anterior tibial muscle) were directly explanted after sacrifice, and RNA was extracted using “RNeasy” Kit (Quiagen, Germany). The RNA concentration and purity were determined photometrically at 260/280 nm. Approximately 80 ng of mRNA was transcribed into cDNA by rt-PCR. In the following nonquantitative PCR the luciferase transcripts were amplified with specific luciferase primers (f 5′ ctg aat aca aat cac aga atc gtc g 3′; r 5′aaa tcc ctg gta atc cgt ttt aga 3′). Additionally the housekeeping gene GAPDH (Glyceraldhyde-3-phosphate-Dehydrogenase) was amplified (f 5′ gca tgt cag atc cac aac gga t 3′; r 5′ tgt cag caa tgc atc ctg ca 3′). All PCR products were detected on 1.5% agarose gel (Serva) with ethidiumbromide (Merck, Germany).

### 2.10. Semiquantitative Analysis

To evaluate the different concentrations of luciferase in the bony specimens at the different time points a semiquantitative PCR with SYBR Green (Biorad, Germany) was performed. SYBR Green binds doublestrand DNA and the resulting DNA-fluorescent-dye-complex absorbs blue light at a wavelength of *λ*_max⁡_ = 498 nm and emits green light at *λ*_max⁡_ = 522 nm. The primers have already been described above. GAPDH served as the control and standard for the Ct value calculation. The amplification of the cDNA was performed with the iCycler (Biorad, Germany). The calculation of the luciferase transcripts was performed using the delta-delta-cycle threshold-method (ΔΔCt). 

### 2.11. Statistics

Comparison of data was performed using one-way ANOVA for independent samples and controlled with Bonferroni correction. Statistical differences were defined at a 95% confidence level. The values are given as mean ± standard deviation. SPSS (SPSS Inc. Chicago, IL USA) software supported statistical evaluation. 

## 3. Results

### 3.1. *In Vitro*

**Cell Culture Luciferase**

**Figure 1:**
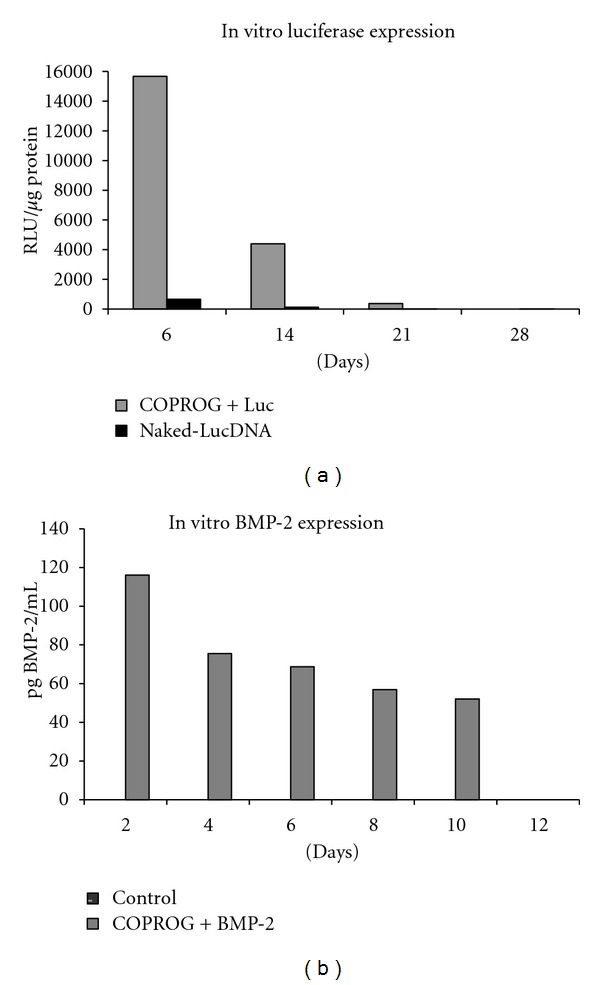
(a) *In vitro* luciferase expression of the COPROG + luciferase versus naked-luciferase DNA group. (b) *In vitro* BMP-2 expression of the COPROG + BMP-2 plasmid versus the control group (copolymer without plasmid).

The results showed a luciferase expression of almost 16,000 relative light units (RLUs) per *μ*g of total protein in the COPROG+luciferase group at day 6 followed by a constant decrease. At day 21 the RLUs were under 400, and at day 28 no RLUs were measurable. In the naked-luciferase DNA group the highest RLU value per *μ*g of protein was reached at day six with 646 RLU/*μ*g. At all other time points a negligible amount was detectable (Figure 1(a)).

The COPROG group showed a high luciferase expression at day six with a constant decrease over time. The naked DNA group showed a 25-fold lower luciferase expression at day six compared to that of the COPROG group, and only negligible amounts were detected in the further time points.

**Cell Culture BMP-2**

The concentration of human BMP-2 in the cell culture supernatant of the COPROG + BMP-2 group was highest at day 2 with 116 pg of BMP-2/mL with a constant decrease at day 4 (75 pg/mL), day 6 (68 pg/mL), day 8 (56 pg/mL), and day 10 (52 pg/mL) to where no BMP-2 was detectable anymore at day 12 (Figure 1(b)).

In the supernatant of the control group no BMP-2 was measured at all time points. The ELISA had no cross reactivity to rodent BMP-2.

The concentration of BMP in the supernatant was highest at day two with a constant decrease over time until day 12. No BMP-2 was measured for the control group at any timepoint.

### 3.2. *In vivo*

**Figure 2:**
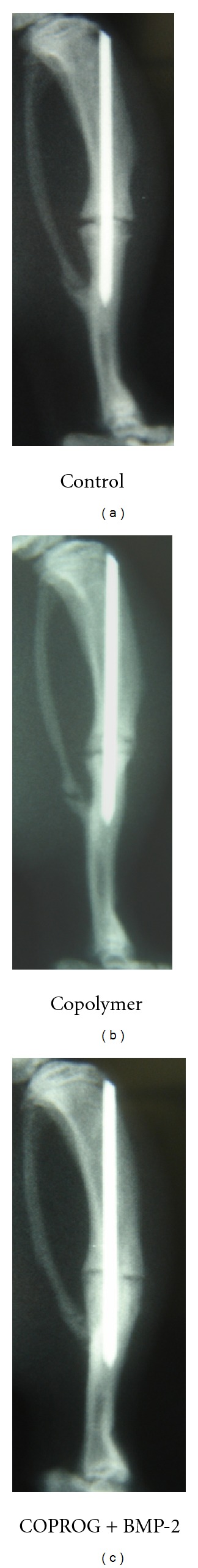
X-ray examination after 42 days (control, copolymer, and COPROG + BMP-2). The X-rays showed no difference in terms of cortical bridging.

The X-ray examinations showed no difference between the groups in terms of cortical bridging (Figure 2). During all examination time points no systemic side effects or relevant changes in blood and serum parameters were seen due to the polymer or the incorporated plasmids and growth factors (results not displayed).

**Biomechanical Testing**

The torsional testing results of the fractured tibiae are displayed as the percentage value of the contralateral unfractured tibae.

**Figure 3:**
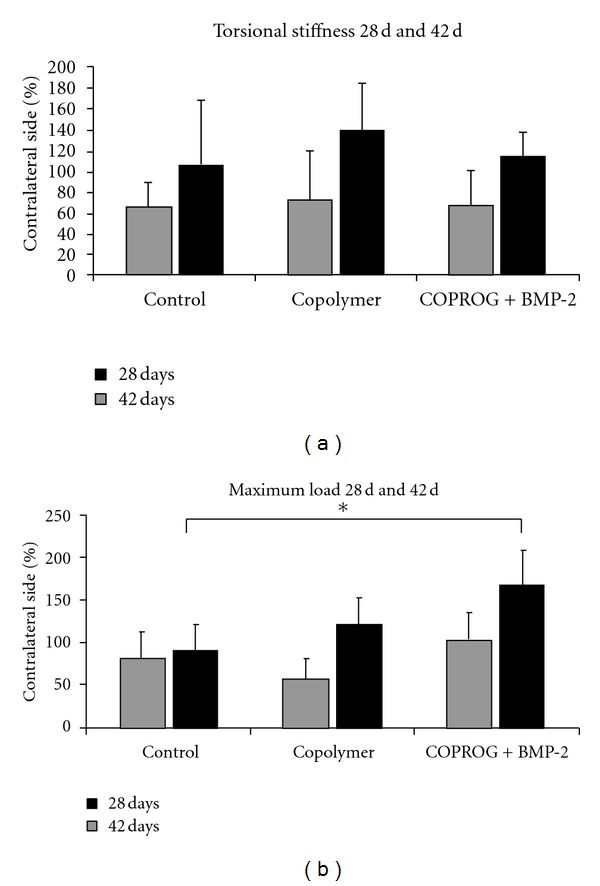
Biomechanical testing (torsional stiffness and maximum load of right rat tibia compared to those of the contralateral side). After 28 and 42 days the COPROG + BMP-2 group showed a significant higher maximum load compared to that of the control group (42 day period). *P < 0.05 (ANOVA, Bonferroni).

After 28 days the highest maximum load was detected for the COPROG + BMP-2 group (III), followed by the control group (I) and the PDLLA + copolymer group (II) (Figure 3). 

Concerning the torsional stiffness the detected values were nearly comparable between the three groups. 

Both the mean maximum load and the torsional stiffness values of the fractured tibiae were below the values of the intact tibiae. Only in the BMP-2 group the maximum load values reached the values of the unfractured tibiae. 

At day 42 after fracture the highest maximum load was detected for the COPROG + BMP-2 group (*P* < 0.05 to control). After 42 days the mean value for the control group was inferior to the copolymer group (Figure 3). The analysis of the torsional stiffness showed comparable results and the same distribution pattern as after 28 days. In both parameters, however, the fractured tibiae reached the value of the unfractured tibiae or even exceeded it.

**Histomorphologic and Histomorphometric Analysis**

**Figure 4:**
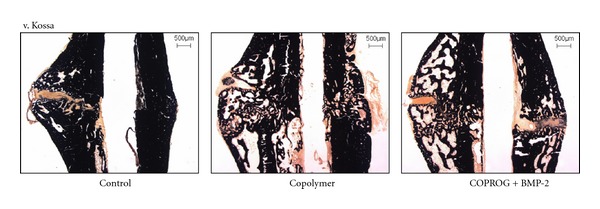
Histological sections of the fracture region (control, copolymer, and COPROG + BMP-2) after 42 days in v. Kossa staining.

The morphology of the callus region did not show significant differences between the three groups. The fracture callus was still composed of fibroblasts and cartilaginous cells after 28 and 42 days. No group showed complete callus consolidation and/or remodelling (Figure 4). A significantly (*P* < 0.05 ANOVA, Bonferroni) less mineralized area in the periosteal callus in the COPROG + BMP-2 group (64.0 ± 11.18%) compared to that of the control group (77.9 ± 6.36%) was found at day 28. This difference was not seen at day 42. No difference was found at both time points for the other parameters including mineralized cortex area, periosteal callus area, periosteal cartilage area (Table 2).

The morphology of the callus region did not show significant differences between the three groups. The fracture callus was composed of fibroblasts and cartilaginous cells after 28 and 42 days. No group showed complete callus consolidation and/or remodelling.

### 3.3. Luciferase RT-PCR

Luciferase RNA was detected in all bone specimens at all time points (2, 4, 7, 28, and 42 days). No transgene luciferase expression could be detected in any other organ at any time point. 

### 3.4. Semiquantitative PCR Analysis

**Figure 5:**
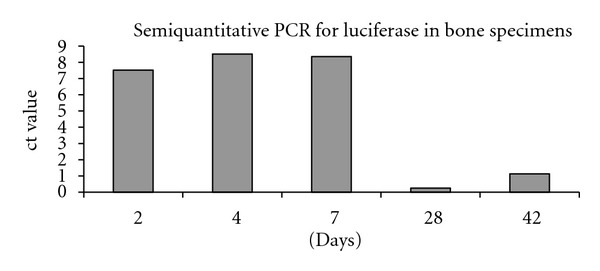
Semiquantitative PCR results of luciferase in bone specimens after 2, 4, 7, 28, and 42 days. Transfection in the bony specimens was highest at day 2, 4, and 7. At days 28 and 42 only low amounts of luciferase were detectable.

After performing semiquantitative PCR analysis transfection in the bony specimens was highest at the early timepoints day 2, 4 and 7. At days 28 and 42 only low amounts of luciferase were detectable (Figure 5).

## 4. Discussion

The incidence of fractures in the United States is 6 million per year from which 1.5 million are long bone fractures. Tibia and fibula fractures have a share of more than one-third on the long bone fractures (approx. 580,000 cases per year) [31]. The socioeconomic consequences of these fractures are immense, and focus is set to improve fracture healing not only under mechanical but also under biological aspects. To merge mechanical stabilization with biological treatment strategies recombinant growth factors like BMP-2 and BMP-7 are additionally applied to mechanical stabilisation devices. So far, the institutional approval for the clinical application of BMP-2 and BMP-7 is restricted to certain fracture or delayed fracture healing conditions [3, 31]. Clinical trials and preclinical studies have both shown a potential for ectopic bone formation as well as edema. These observations might partly be attributed to the collagen carriers used to deliver BMP, which have been hypothesized to be not optimal protein delivery systems [32, 33]. The important issue, when applying growth factors is to achieve constant and lasting levels of the protein at the application site to ensure the desired osteoinductive effect. So there is a clear need for controlled drug delivery systems, and a promising application method might be a gene therapeutic approach. 

The present study introduces a nonviral BMP-2 plasmid application for the stimulation of fracture healing. A newly developed gene vector based on the polymeric encapsulation of plasmids was used [26]. The gene binding and condensation capacities as well as the *in vitro* and *in vivo* transfection properties have been described before [24, 25]. The *in vitro* studies proved the transfection of cells by the use of the COPROG method. Primary rat osteoblast like cells expressed both, the marker luciferase, and also the therapeutical protein human BMP-2 over a time period of minimally 10 days. In this study the proof of concept was then extended to the *in vivo* fracture healing model. Besides the effect on bone healing the possible systemic distribution was investigated. There was a stimulating effect on fracture healing, which might be more effective using a higher plasmid concentration. A time-dependent expression of the reporter gene was detectable in the fractured bone, but no expression was detectable in the contralateral bone or in other investigated tissues. Therefore, these results indicate that the *in vivo* transfection of cells within the fracture region is possible. Important for the safety issue, no systemic transfection was detectable. 

A possible explanation for the weak effect of the nonviral gene therapy might be the lower transfection rate compared to that of existing viral methods. It is known that transfection efficiency is generally lower for nonviral vectors compared to that of viral vector systems. Franceschi et al. estimated that the cellular uptake of nonviral vectors into the cell is to be 10^9^ less than that of viral vectors [34]. The *in vitro* studies revealed that a 40 *μ*g dose of luciferase DNA was enough to transfect cells for a period from 2 until 21 days. Applying the BMP-2 plasmid per COPROG *in vitro* the transfection lasted at least for 10 days. These results are in accordance with previous work, where COPROG formulations were combined with a fibrin glue composition instead of PDLLA, and transfection of human keratinocytes and rabbit articular chondrocytes was tested [24]. For naked DNA compositions the luciferase reporter gene measurement was only present at day 6, whereas the COPROG formulation showed a transfection of human keratinocytes with an initial peak at day one and sharply declining during the measurement period which ended at day 13. The luciferase expression in rabbit articular chondrocytes persisted for at least 21 days (end of measurement) with a peak on day 3 followed by a sharp decline until day 7. 

In another study from Scherer et al. [25] carrier-mediated gene delivery was compared to standard vectors-in a vector loaded collagen sponge model. Collagen sponges were loaded with either naked DNA, PEI-DNA, or copolymer-protected PEI-DNA formulations with a DNA dose of approximately 50 *μ*g for each application. In the cell culture study using NIH 3T3 mouse fibroblasts a luciferase expression could be observed until day 7 for the naked DNA. The copolymer-protected PEI-DNA showed a reporter gene expression throughout the experimental period of 56 days, and the level of expression was up to a 100-fold higher than with PEI DNA and up to several 100-folds higher than with naked DNA. This is not surprising because it was already shown that unprotected DNA is rapidly degraded in an *in vivo* situation [35]. 

In our study comparable results were obtained. A relevant luciferase expression was detectable until day 21 in the copolymer protected vector group. This was up to 25-fold higher than that of the naked DNA group. The difference in expression kinetics between the studies is probably due to different rates of vector uptake and the general differences in cell physiology and is commonly seen in gene delivery. 

For *in vivo* testing Scherer et al. implanted the vector-loaded collagen sponges subcutaneously into male wistar rats [25]. Luciferase activity of the copolymer-protected vector group was present after 3 and 7 days and vanished after 14 days, whereas no luciferase could be measured in the naked DNA group at all time points. This is in accordance to our *in vivo* findings. The quantitative PCR showed the highest transgene expression in the bone specimens up to day 7 with a decrease until day 42. 

But since transgene expression lasted until day 42 the humble effect on bone formation may be due to the low concentration of the plasmid, because only 40 *μ*g of DNA was used per application. Clinically, recombinant BMP is being administered at doses that are million times greater than its normal concentration in bone, and there are concerns about both the safety and the cost of such supraphysiologic doses [32]. For example, 1 kg of human bone yields 1 *μ*g of BMP. One vial of OP-1 (rhBMP-7) contains 3.5 mg, and this amount is equivalent to all the BMP-7 in the entire skeletons of two people.

The dosage of plasmid DNA we used for this study seems fairly low compared to that of other studies. In a study of Fang et al. [36] 0.5–1.0 mg of plasmid DNA coding for BMP-4 and parathyroid hormone with a collagen sponge carrier was used to bridge a tibial critical bone defect in a rat model. Cortical bridging occurred after a 4-week period, but the DNA dose was a 10–25-fold higher than that in our study.

Bonadio et al. used doses from 1 mg up to 100 mg of plasmid-DNA which encoded for a secreted fragment of human parathyroid hormone to fill critical size defects in a canine bone defect model [7]. Defects treated with up to 20 mg of plasmid-DNA showed no effect, whereas 40 mg was able to fill 25% of the defect after 4 weeks. Even the doses of 100 mg of plasmid-DNA were not capable to fully complete the gap filling, they led to a filling of approximately. 75% after 6 weeks.

Exhausting the portion of delivered DNA by either escalating the utilized amount or using highly capable transfection systems might lead to safety concerns in terms of systemic distribution and infammatory reactions. In a study 1 × 10^11^ particles of an adenoviral vector were injected into an iliac crest defect of sheep, and luciferase activity was measured. High concentrations of luciferase were seen in bone, the adjacent muscle, and even the surrounding soft tissue up to 5 weeks. But also low concentrations of luciferase were detected in the kidney and the thyroid gland at early time points as an indicator for a low systemic distribution [12]. Ishihara et al. reported about an increased serum antiadenoviral vector antibody after injecting 5 × 10^11^ adenoviral particles into a metatarsal osteotomy gap of mature horses. Neutralizing antibodies were found in the serum throughout the whole experimental period (2, 4, and 6 weeks) [37]. 

In a rabbit study 1 × 10^10^ adenoviral particles with cDNA encoding for luciferase were injected into a segmental femoral defect. For the safety aspect tissues like spleen, liver, lung as well as bone, bone marrow and surrounding muscle from the contralateral femoral diaphysis were tested for luciferase expression. The results showed a slight luciferase activity until day 5 in the liver tissue, which was assessed to be due to the fact that a minor fraction of the vector circulated systemically [38]. To limit the possibility of a systemic distribution of genetic material we were interested in an alternative approach to achieve local gene delivery with fairly low DNA doses. The RT-PCR analysis in our study showed a thorough transgene expression in all bony specimens at all time points, whereas no transgene product was detected in other distant or even local tissues like the surrounding muscle. Also Scherer et al., when subcutaneously implanting 50 *μ*g of DNA in form of COPROG collagen sponges into rats, could not find any systemic distribution. Distant organs like heart, lung, liver, spleen, and kidney were tested negative for transgene expression. 

Favourable about this *in vivo* concept of growth-factor-specific gene delivery is the simple technique of preparation and application, since the implant serves as fracture stabilization device on the one hand and on the other hand as the carrier for the nonviral gene delivery system. No other invasive treatments like opening fracture sites or injection treatments are necessary in order to deliver the genes to the site of interest. In a previous study we already investigated the effect of recombinant human BMP-2 application using the similar coating technique and fracture model [39]. The results showed a significantly elevated maximum load and torsional stiffness after 28 and 42 days compared to those of the controls. The biomechanical findings where supported by the histological data, where the rhBMP group was significantly superior in terms of callus remodelling. Interestingly the absolute value of the maximum load after 42 days for the COPROG-BMP-2 group in the presented study was even higher than in the rhBMP-2 group of the previous study, whereas neither the other biomechanical, nor the histological investigations could back up this result. The reason for that remains somewhat unclear, especially since transgene expression was measured throughout the whole experimental period. But then, transgene expression alone is not able to predict the therapeutic benefit of a gene therapy approach. An important issue might be the responsiveness of the BMP signalling pathway of each individual. And it is still a major issue to find out how long transgene expression should take place. The ideal cut has to be found between too short which might not lead to insufficient bone formation and too long which could lead to an unwanted excessive callus formation or even exceed the boundaries of a local therapy at the desired regeneration site and causing systemic side effects. But this timepoint has to be found for each individual vector formulation together with the adequate amount of DNA. More information is needed concerning the right amount of DNA and vector dose, which will lead to an adequate and safe reaction in the specific environment of a fracture healing situation. 

## 5. Conclusions

For the first time, a nonviral BMP-2-plasmid application from COPROG-coated implants showed an effect on fracture healing *in vivo*. The implant served as fracture stabilization device and as a plasmid delivery system. A positive effect on fracture healing could be shown in this study; however, the effect of the COPROG vector may be improved with higher plasmid concentrations. While transfection stayed local, and no biodistribution in distant organs could be detected; the vector system was considered to be safe in this study. Since this is the first *in vivo* study using the COPROG vector for fracture repair, there is an ample scope to improve the efficacy by charging the COPROGs with a higher plasmid concentration, and further studies will follow to solve this equation. 

## Figures and Tables

**Table 1:** Investigated groups and timepoints of measurements.

Group	Implant	Analysis	2d	4d	7d	28d	42d
I	K-wire control (no coating)	(a) Biomechanical testing				*n* = 10	*n* = 10
		(b) Histology				*n* = 10	*n* = 10
II	+ PDLLA + copolymer	(a) Biomechanical testing				*n* = 10	*n* = 10
		(b) Histology				*n* = 10	*n* = 10
III	+ PDLLA + COPROG + BMP-2 (40 *μ*g)	(a) Biomechanical testing				*n* = 10	*n* = 10
		(b) Histology				*n* = 10	*n* = 10
IV (safety)	+ PDLLA + COPROG + Luc (40 *μ*g)	(c) Luciferase PCR	*n* = 5	*n* = 5	*n* = 5	*n* = 5	*n* = 5

**Table 2:** Histomorphometric analysis of the tibial callus region after 28 and 42 days. There was significantly less mineralized area in the periosteal callus area in the COPROG + BMP-2 group compared to the control group at day 28 and no difference in the other parameters between the groups at both time points.

	28 days			42 days		
	Control	Copolymer	COPROG + BMP	Control	Copolymer	COPROG + BMP
Mineralized Ar./Co.Ar (%)	97.9 ± 1.32	97.4 ± 0.97	95.4 ± 1.49	97.2 ± 0.87	97.2 ± 0.92	97.1 ± 1.19
Periosteal Cl.Ar (Ti.Dm) (mm)	5.0 ± 0.88	5.2 ± 0.93	5.5 ± 1.86	5.0 ± 1.26	6.4 ± 1.78	6.8 ± 2.38
Mineralized Ar./Ps.Cl.Ar (%)	77.9 ± 6.36	71.4 ± 6.39	64.0 ± 11.18^1^	79.4 ± 9.28	73.9 ± 9.31	71.0 ± 11.64
Cartilage Ar./Ps.Cl.Ar (%)	5.3 ± 4.18	9.6 ± 5.21	5.1 ± 3.59	6.0 ± 4.54	6.3 ± 3.34	5.3 ± 4.65

*P* < 0.05 (ANOVA, Bonferroni).

^1^Significant difference to Control group at the same time point.
